# Mental Health Literacy in Foundation Doctors - a Survey in the York and Humber Area

**DOI:** 10.1192/bjo.2022.161

**Published:** 2022-06-20

**Authors:** Ioana Varvari, Harry Foster, Pratibha Nirodi

**Affiliations:** 1 Tees, Esk and Wear Valley NHS Foundation Trust, York, United Kingdom; 2 Leeds and York Partnership NHS Foundation Trust, Leeds, United Kingdom; 3 Tees, Esk and Wear Valley NHS Foundation Trust, Harrogate, United Kingdom; 4 Health Education England, York and Humber, United Kingdom

## Abstract

**Aims:**

We aimed to measure the baseline mental health literacy in Foundation Doctors in the Yorkshire and Humber area, identify any gaps in knowledge with the purpose of addressing these within the new foundation psychiatry teaching program, developed by North Yorkshire Health Education England.

**Methods:**

In January 2021, a questionnaire comprising of O'Connor's Mental Health Literacy Scale was sent electronically to all Foundation Doctors in the York and Humber area, that were in a placement at that time. The O'Connor's Mental Health Literacy Scale (MHLS) has been used since its publication in 2015 and is a 35 item, univariate scale that demonstrated good internal and test-retest reliability. It covers the following attributes: a) ability to identify disorders, b) knowledge about seeking information, risk factors and etiology, self-treatment, resources and support available, c) attitudes about mental disorders and seeking professional help. The anonymized data were collected and analysed in Microsoft Excel.

**Results:**

In total, we received 49 responses to the questionnaire. Overall, 85% of respondents demonstrated good mental health literacy. Breaking this down further, 91% demonstrated knowledge of core psychiatric diagnostic criteria, 68.4% were literate in etiology and risk factors, 92% and respectively 95.9% understand what resources for treatment and professional help are available. Importantly when looking at attitudes about mental disorders overall 17% of respondents showed a degree of stigma and barriers in seeking professional help. For example, 2% strongly agreed that mental health conditions are not real illnesses, 34.7% were unsure whether people with mental illness are dangerous, 40.9% neither agreed nor disagreed they would move next door with someone with a mental illness and 14.3% would not be willing to have someone with a mental illness marrying into the family. When looking at barriers to seeking help, 12% answered they would not tell someone if they had a mental health problem, with 16.3% unsure whether they would tell someone if they had a mental health problem.

**Conclusion:**

Overall, our survey demonstrated good mental health literacy in our cohort, however, there are areas of improvement, the main ones being etiology, risk factors, and attitudes towards mental health. It is important to recognize these deficits, as they have been linked with poor health outcomes and barriers in seeking and providing care. Moving forward, standardization of teaching programs and anti-stigma training could be an evidence-based approach to tackling these issues.

